# Incidence and risk factors of cardiovascular mortality in patients with gastrointestinal adenocarcinoma

**DOI:** 10.1371/journal.pone.0262013

**Published:** 2023-01-27

**Authors:** Nso Nso, Akwe Nyabera, Mahmoud Nassar, Yolanda Mbome, Kelechi Emmanuel, Mohsen Alshamam, Vickram Sumbly, Laura Guzman, Tanveer Shaukat, Rubal Bhangal, Gilbert Ako Ojong, Farshid Radparvar, Vincent Rizzo, Most Sirajum Munira

**Affiliations:** 1 Department of Medicine, Icahn School of Medicine at Mount Sinai / NYC H&H Queens, New York, NY, United States of America; 2 Department of Medicine, Richmond University Medical center, Staten Island, NY, United States of America; 3 Department of Medicine, University of Pittsburgh Medical Center Pinnacle, Harrisburg, PA, United States of America; 4 Department of Medicine, La Magna Health/United Regional Hospital, Wichita Falls, Texas, United States of America; 5 Division of Cardiology, Icahn School of Medicine at Mount Sinai / NYC H&H Queens, New York, NY, United States of America; Federation University Australia, AUSTRALIA

## Abstract

**Background:**

Gastrointestinal (GI) cancers are common and fatal. Improved cancer-directed therapies, with thier substantial role in improving cancer-specific survival, may increase non-cancer mortality−including cardiovascular mortality−in these patients.

**Aim:**

To identify the risk factors of cardiovascular mortality in GI adenocarcinoma patients.

**Methods:**

Data of GI adenocarcinoma patients were gathered from the Surveillance, Epidemiology, and End Results database. We used Pearson’s chi-square test to assess the relationships between categorical variables. We used the Kaplan-Meyer test in the univariate analysis and Cox regression test for the multivariate analysis.

**Results:**

Among 556,350 included patients, 275,118 (49.6%) died due to adenocarcinoma, 64,079 (11.5%) died due to cardiovascular causes, and 83,161 (14.9%) died due to other causes. Higher rates of cardiovascular mortality were found in patients ≥ 50 years (HR, 8.476; 95% CI, 7.91–9.083), separated (HR, 1.27; 95% CI, 1.184–1.361) and widowed (HR, 1.867; 95% CI, 1.812–1.924), patients with gastric (HR, 1.18; 95% CI, 1.1–1.265) or colorectal AC (HR, 1.123; 95% CI, 1.053–1.198), and patients not undergone surgery (HR, 2.04; 95% CI, 1.958–2.126). Lower risk patients include females (HR, 0.729; 95% CI, 0.717–0.742), blacks (HR, 0.95; 95% CI, 0.924–0.978), married (HR, 0.77; 95% CI, 0.749–0.792), divorced (HR, 0.841; 95% CI, 0.807–0.877), patients with pancreatic AC (HR, 0.83; 95% CI, 0.757–0.91), and patients treated with chemotherapy (HR, 0.416; 95% CI, 0.406–0.427).

**Conclusions:**

Risk factors for cardiovascular mortality in GI adenocarcinoma include advanced age, males, whites, separated and widowed, gastric or colorectal adenocarcinoma, advanced grade or advanced stage of the disease, no chemotherapy, and no surgery. Married and divorced, and patients with pancreatic adenocarcinoma have a lower risk.

## 1. Introduction

Gastrointestinal (GI) cancers constitute a major part of cancer cases and cancer-related deaths [1]. Most GI cancers has a poor prognosis because they are usually diagnosed at a late stage [2, 3]. In 2020, GI cancers caused more than 3.5 million deaths and 5 million new cancer cases [4]. Colorectal cancer (CRC) is the most common GI cancer and the third most common cancer of all organs. It caused more than 1.9 million new cases and 935,000 deaths in 2020 [4]. Its incidence rises in countries with high human development index [5]. Risk factors for CRC include older age, family history, alcohol consuming, smoking, obesity, lack of exercise, and some dietary factors including red and processed meat, while suggested protective dietary factors include fruits and vegetables containing natural fibers [6, 7].

Other common GI cancers include stomach, liver, esophageal, and pancreatic cancers. Gastric cancer is the fifth most common and fourth-most fatal malignancy [4]. Risk factors for stomach cancer include male sex, H. pylori infection, tobacco consumption, and atrophic gastritis [8, 9].

Primary liver cancer is the sixth among cancers regarding incidence and the third regarding mortalities [4]. The two commonest types of liver cancer are hepatocellular carcinoma (HCC) and intrahepatic bile duct cancers (cholangiocarcinoma) [4]. The risk factors for liver cancer include hepatitis B and hepatitis C virus infections, male sex, chronic liver disease, alcohol consumption, smoking, type 2 diabetes, liver cirrhosis, and aflatoxin-contaminated foods [4, 10–12]. Esophageal cancer is the seventh most common cancer and the sixth in cancer-caused mortalities. Its two main histological types, squamous cell carcinoma (SCC) and adenocarcinoma (AC), vary in their geographic distribution and risk factors [4]. The esophagus’s adenocarcinoma usually affects the lower part of the esophagus and the gastroesophageal junction [13]. It is less common than squamous cell carcinoma, but it surpasses SCC in western countries and has higher mortality rates [4, 13, 14]. Risk factors for esophageal adenocarcinoma include chronic gastroesophageal reflux, Barret’s esophagus, and obesity [14].

Generally, cancer death rates have declined, and rates of survival have increased due to advances in cancer therapies [15, 16]. This improvement of survival may increase the incidence of non-cancer—including cardiovascular—mortality [17, 18]. Assessment and monitoring of cardiovascular risk in cancer patients should start before cancer treatment to decrease the burden of cardiovascular diseases in these patients [19]. Monitoring should continue during and after treatment to detect expected cardiotoxic treatment side effects [19, 20]. Identification of patients at higher cardiovascular risk is important for effective control of morbidity and mortality [19]. Ignoring cardiovascular monitoring and cardiovascular side effects may cause serious conditions (as stroke and myocardial infarctions) that prevent continuation of optimal cancer therapy [19]. Many cancer therapies—including radiation and chemotherapeutics—are considered as cardiovascular risk factors [19, 21–23]. So, cardiovascular assessment and cardiovascular disease prevention in cancer patients should start at cancer diagnosis, continue while they are receiving their therapy, and continue through their lifetime [19].

There is little information in the literature about cardiovascular mortality in patients with GI adenocarcinomas. The risk factors of cardiovascular mortality in these patients need to be identified. In this retrospective study, we aim to identify the incidence and the risk factors associated with cardiovascular mortality in patients with GI adenocarcinomas registered in the Surveillance, Epidemiology and End Results (SEER) database.

## 2. Methods

### 2.1. Study population and study design

In this retrospective cohort study, we collected data of the patients with GI adenocarcinoma from the SEER database, which holds data of cancer patients in the United States and is an authoritative source for cancer statistics. We extracted data using the SEER*Stat (version 8.3.8) and included patients from 1975 to 2015. Patients were followed up until death or until 2016.

### 2.2. Ethics statement

Data registered in the SEER database is freely released and available upon requesting access to the database. Besides, cancer is reportable in the United States. So, this study did not require informed consents from patients whose data were used in the study.

### 2.3. Data collection

Data of the dependent and the independent variables were extracted from the SEER program. Dependent variables of interest included: 1) CV mortality (mortality due to aortic aneurysm and dissection, atherosclerosis, cerebrovascular diseases, diseases of the heart, hypertension, and other diseases of arteries, arterioles, or capillaries). 2) Adenocarcinoma mortality. 3) Other mortalities. Independent variables included age, sex, race, marital status, primary site of the disease [esophagus, stomach, small intestine, colon and/or rectum, anus, liver, pancreas and GI tract not otherwise specified (NOS)], stage of the disease (localized, regional, distant and unstaged), grade of the cancer, chemotherapy, radiation therapy and cancer directed surgery.

### 2.4. Statistical analysis

All analyses in this study were conducted using SPSS software for windows (version 26.0). We assessed the relationships between categorical variables using Pearson’s chi square test [24]. To assess the effect of the independent variables on the overall, cardiovascular-specific and adenocarcinoma-specific survival months; we conducted a univariate analysis using the Kaplan-Meyer test [25]. We presented data of the univariate analysis as median (months) and 95% confidence interval (CI). To assess the effect of the independent variables on the overall, cardiovascular-specific and adenocarcinoma-specific mortality; we conducted a multivariate analysis using the Cox regression test [25]. We presented data of the multivariate analysis as hazard ratio (HR) and 95% CI. To assess the probability of cardiovascular diseases (CVDs) in the total study population, we used the binary logistic regression analysis [26] and presented data as odds ratio (OR) and 95% CI. In any analysis, we considered the effect significant when P-value is less than 0.05.

## 3. Results

### 3.1. Study population characteristics

We included 556,350 GI adenocarcinoma patients in our study. The mean age at diagnosis of the included participants was 67.41 ± 12.66 years, and 54% of the study population are males. Of the included participants, a total of 408,675 (73.4%) participants died: 261,435 (47%) died due to adenocarcinoma, 64,079 (11.5%) died due to CV causes, and 83,161 (14.9%) died due to other causes. Heart diseases made up the majority of CV causes of mortality (49,058 patients, 76.6% of CV mortalities, and 8.8% of the total study population). Colorectal AC patients comprised 76.1% of included participants, followed by gastric (9%), pancreatic (6.2%), esophageal (4.1%), hepatobiliary (3.5%), small intestinal (0.9%), GI tract NOS (0.1%) and anal AC (0.1%). Table 1 shows the details of clinicopathologic characteristics of the total study population and of each studied mortality-specific group.

**Table 1:** Clinicopathologic features of gastrointestinal adenocarcinoma patients.

Variables	Total (n = 556350)	Survivors (n = 147675)	Adenocarcinoma mortality (n = 261435)	Cardiovascular mortality (n = 64079)	Aortic aneurysm and dissection (n = 866)	Atherosclerosis (n = 1196)	Cerebrovascular deaths (n = 10581)	Diseases of the heart (n = 49058)	Hypertension (n = 1698)	Other diseases of arteries, arterioles, or capillaries (n = 680)	Other mortalities (n = 83161)
Age	67.41 (12.66)	61.65 (12.517)	67.58 (12.59)	74.65 (9.3)	73.25 (8.829)	77.79 (7.72)	74.86 (9.102)	74.57 (9.348)	74.59 (9.5)	72.91 (10.07)	71.53 (10.66)
Sex											
Male	300227 (54%)	76962 (52.1%)	145926 (55.8%)	32709 (51%)	529 (61.1%)	537 (44.9%)	4630 (43.8%)	25944 (52.9%)	728 (42.9%)	341 (50.1%)	44630 (53.7%)
Female	256123 (46%)	70713 (47.9%)	115509 (44.2%)	31370 (49%)	337 (38.9%)	659 (55.1%)	5951 (56.2%)	23114 (47.1%)	970 (57.1%)	339 (49.9%)	38531 (46.3%)
Race											
White	454131 (81.6%)	116546 (78.9%)	212210 (81.2%)	54920 (85.7%)	742 (85.7%)	1105 (92.4%)	8957 (84.7%)	42204 (86%)	1337 (78.7%)	575 (84.6%)	70455 (84.7%)
Black	55268 (9.9%)	14563 (9.9%)	28161 (10.8%)	5330 (8.3%)	46 (5.3%)	51 (4.3%)	828 (7.8%)	4100 (8.4%)	232 (13.7%)	73 (10.7%)	7214 (8.7%)
Others	46951 (8.4%)	16566 (11.2%)	21064 (8.0%)	3829 (6%)	78 (9%)	40 (3.3%)	796 (7.5%)	2754 (5.6%)	129 (7.6%)	32 (4.7%)	5492 (6.6%)
Marital status											
Single	68355 (12.3%)	21063 (14.3%)	33027 (12.6%)	5790 (9%)	51 (5.9%)	98 (8.2%)	854 (8.1%)	4559 (9.3%)	169 (10%)	59 (8.7%)	8475 (10.2%)
Married	323556 (58.2%)	95479 (64.7%)	148995 (57%)	32932 (51.4%)	510 (58.9%)	544 (45.5%)	5452 (51.5%)	25270 (51.5%)	814 (47.9%)	342 (50.3%)	46150 (55.5%)
Separated	6703 (1.2%)	1450 (1%)	3262 (1.2%)	937 (1.5%)	15 (1.7%)	18 (1.5%)	146 (1.4%)	712 (1.5%)	30 (1.8%)	16 (2.4%)	1054 (1.3%)
Divorced	44512 (8%)	13321 (9%)	21533 (8.2%)	3579 (5.6%)	51 (5.9%)	45 (3.8%)	542 (5.1%)	2792 (5.7%)	99 (5.8%)	50 (7.4%)	6079 (7.3%)
Widowed	113224 (20.4%)	16362 (11.1%)	54618 (20.9%)	20841 (32.5%)	239 (27.6%)	491 (41.1%)	3587 (33.9%)	15725 (32.1%)	586 (34.5%)	213 (31.3%)	21403 (25.7%)
Primary site											
Esophagus	22635 (4.1%)	3161 (2.1%)	16457 (6.3%)	1063 (1.7%)	21 (2.4%)	19 (1.6%)	135 (1.3%)	864 (1.8%)	17 (1%)	7 (1%)	1954 (2.3%)
Gastric	50017 (9%)	5881 (4%)	35192 (13.5%)	3653 (5.7%)	51 (5.9%)	75 (6.3%)	571 (5.4%)	2812 (5.7%)	106 (6.2%)	38 (5.6%)	5291 (6.4%)
Small intestine	5031 (0.9%)	1025 (0.7%)	3010 (1.2%)	316 (0.5%)	4 (0.5%)	5 (0.4%)	57 (0.5%)	233 (0.5%)	13 (0.8%)	4 (0.6%)	680 (0.8%)
Colorectal	423256 (76.1%)	133033 (90.1%)	162808 (62.3%)	57083 (89.1%)	766 (88.5%)	1060 (88.6%)	9482 (89.6%)	43658 (89%)	1516 (89.3%)	601 (88.4%)	70332 (84.6%)
Anal	584 (0.1%)	155 (0.1%)	308 (0.1%)	50 (0.1%)	(%)	1 (0.1%)	9 (0.1%)	36 (0.1%)	2 (0.1%)	2 (0.3%)	71 (0.1%)
Hepato-biliary	19449 (3.5%)	2751 (1.9%)	13190 (5%)	1011 (1.6%)	15 (1.7%)	21 (1.8%)	156 (1.5%)	776 (1.6%)	30 (1.8%)	13 (1.9%)	2497 (3%)
Pancreatic	34744 (6.2%)	1655 (1.1%)	30143 (11.5%)	882 (1.4%)	9 (1%)	15 (1.3%)	168 (1.6%)	663 (1.4%)	13 (0.8%)	14 (2.1%)	2064 (2.5%)
GIT NOS	634 (0.1%)	14 (0.01%)	327 (0.1%)	21 (0.03%)	(%)	(%)	3 (0.03%)	16 (0.03%)	1 (0.1%)	1 (0.1%)	272 (0.3%)
Stage											
Localized	165218 (29.7%)	65072 (44.1%)	35915 (13.7%)	28994 (45.2%)	407 (47%)	541 (45.2%)	4814 (45.5%)	22116 (45.1%)	823 (48.5%)	293 (43.1%)	35237 (42.4%)
Regional	234702 (42.2%)	70606 (47.8%)	100025 (38.3%)	28119 (43.9%)	371 (42.8%)	520 (43.5%)	4811 (45.5%)	21384 (43.6%)	718 (42.3%)	315 (46.3%)	35952 (43.2%)
Distant	136467 (24.5%)	10107 (6.8%)	112718 (43.1%)	4380 (6.8%)	57 (6.6%)	83 (6.9%)	602 (5.7%)	3488 (7.1%)	99 (5.8%)	51 (7.5%)	9262 (11.1%)
Unstaged	19963 (3.6%)	1890 (1.3%)	12777 (4.9%)	2586 (4%)	31 (3.6%)	52 (4.3%)	354 (3.3%)	2070 (4.2%)	58 (3.4%)	21 (3.1%)	2710 (3.3%)
Grade											
I	51177 (9.2%)	12761 (8.6%)	20656 (7.9%)	8155 (12.7%)	135 (15.6%)	142 (11.9%)	1320 (12.5%)	6273 (12.8%)	192 (11.3%)	93 (13.7%)	9605 (11.5%)
II	350454 (63%)	108400 (73.4%)	144129 (55.1%)	42911 (67%)	549 (63.4%)	795 (66.5%)	7094 (67%)	32889 (67%)	1154 (68%)	430 (63.2%)	55014 (66.2%)
III	146479 (26.3%)	24578 (16.6%)	91899 (35.2%)	12367 (19.3%)	172 (19.9%)	244 (20.4%)	2055 (19.4%)	9414 (19.2%)	331 (19.5%)	151 (22.2%)	17635 (21.2%)
IV	8240 (1.5%)	1936 (1.3%)	4751 (1.8%)	646 (1%)	10 (1.2%)	15 (1.3%)	112 (1.1%)	482 (1%)	21 (1.2%)	6 (0.9%)	907 (1.1%)
Chemotherapy											
No/Unknown	352281 (63.3%)	81315 (55.1%)	150285 (57.5%)	54843 (85.6%)	748 (86.4%)	1092 (91.3%)	9128 (86.3%)	41895 (85.4%)	1407 (82.9%)	573 (84.3%)	65838 (79.2%)
Yes	204069 (36.7%)	66360 (44.9%)	111150 (42.5%)	9236 (14.4%)	118 (13.6%)	104 (8.7%)	1453 (13.7%)	7163 (14.6%)	291 (17.1%)	107 (15.7%)	17323 (20.8%)
Radiation therapy											
No radiation	459918 (82.7%)	119441 (80.9%)	208886 (79.9%)	58533 (91.3%)	797 (92%)	1126 (94.1%)	9792 (92.5%)	44687 (91.1%)	1529 (90%)	602 (88.5%)	73058 (87.9%)
Beam radiation	94160 (16.9%)	27640 (18.7%)	51188 (19.6%)	5439 (8.5%)	68 (7.9%)	68 (5.7%)	774 (7.3%)	4288 (8.7%)	165 (9.7%)	76 (11.2%)	9893 (11.9%)
Other types of radiation	2272 (0.4%)	594 (0.4%)	1361 (0.5%)	107 (0.2%)	1 (0.1%)	2 (0.2%)	15 (0.1%)	83 (0.2%)	4 (0.2%)	2 (0.3%)	210 (0.3%)
Surgery performed											
Yes	450102 (80.9%)	141630 (95.9%)	176026 (67.3%)	58501 (91.3%)	801 (92.5%)	1095 (91.6%)	9815 (92.8%)	44596 (90.9%)	1560 (91.9%)	634 (93.2%)	73945 (88.9%)
No	106248 (19.1%)	6045 (4.1%)	85409 (32.7%)	5578 (8.7%)	65 (7.5%)	101 (8.4%)	766 (7.2%)	4462 (9.1%)	138 (8.1%)	46 (6.8%)	9216 (11.1%)

GIT = gastrointestinal tract. NOS = not otherwise specified. All data are presented as number (percentage) except age presented as mean (standard deviation).

### 3.2. Univariate analysis of overall survival, cardiovascular-specific survival, and adenocarcinoma-specific survival

The median overall survival was equal for patients < 50 years and for patients ≥ 50 years (19 months). It was higher in females (20 months) than in males (19 months). It was higher in white race (20 months) than in black (16 months) and other races (19 months). It was higher in married patients (22 months) than in single (15 months), separated (20 months), divorced (17 months) and widowed patients (17 months). As expected, it was inversely related to tumor grade (28 months with grade I and 8 months in grade IV) and tumor stage (52 months in localized and 7 months in distant and in unstaged tumor). It was higher in patients treated with surgery (30 months) than patients with no cancer-directed surgery (4 months). It was lower in patients treated with chemotherapy (19 months) than those who were not (20 months). It was similar in patients treated with beam radiation and those not treated by radiation (19 months), but it was lower in patients treated with other types of radiation (18 months). Regarding the site of AC; the median overall survival was higher in colorectal (30 months) than anal (19 months), esophageal, small intestinal (9 months), gastric, hepatobiliary (8 months), pancreatic (5 months), and non-specified GI adenocarcinoma (1 month).

The median cardiovascular-specific survival was higher in pateints < 50 years than in patients ≥ 50 years (108 vs 62 months), in females than in males (67 vs 57 months), in other races than in white and black (65 vs 63 and 44 months, respectively), in married than in single, separated, divorced, and widowed patients (75 vs 46, 64, 53, and 50 months, respectively). It was inversely related to the tumor grade (73 months in grade I vs 35 months in grade IV) and stage (74 months in localized vs 6 months in distant). It was higher in patients treated with surgery than not treated (70 vs 4 months), and in patients not treated with radiotherapy than treated with beam or other types of radiation (63 vs 55 and 44 months, respectively), while the effect of chemotherapy was insignificant (65 vs 61 months, P = 0.063). Regarding the site of AC; colorectal AC had the longest median CV-specific survival (68 months), followed by small intestinal, gastric, anal, hepatobiliary, esophageal and pancreatic AC (28, 22, 20, 15, 12 and 3 months, respectively).

Median AC specific survival was higher in patients < 50 years than in patients ≥ 50 years (17 vs 12 months), in males than in females (13 vs 12 months), in other races than in white and black races (14 vs 12 months), in married than in single, separated, divorced, and widowed (14 vs 11, 13, 12 and 9 months, respectively). It was inversely related to the tumor stage (25 months in localized vs 7 months in distant), but its relation with the tumor grade was not regular (higher in grade II than in grades I, III and IV; 17 vs 15, 8 and 7 months, respectively). It was higher in patients treated with surgery than not (19 vs 4 months), in patients treated with chemotherapy than not (16 vs 9 months), and in patients treated with beam radiation than in patients treated with other types of radiation or not treated with radiation (16 vs 15 and 11 months, respectively).

Table 2 shows the details of the univariate analysis results. S1 File shows the survival curves for cancer-specific and for cardiovascular-specific mortalities with different variables.

**Table 2:** Univariate analysis using Kaplan Meier test.

Variables	Adenocarcinoma survival months, median (95% CI)	P-value	Cardiovascular survival months, median (95% CI)	P-value	Total survival months, median (95% CI)	P-value
Age		< 0.001		< 0.001		< 0.001
< 50 years	17 (16.677–17.323)		108 (94.915–121.085)		19 (18.646–19.354)	
≥ 50 years	12 (11.91–12.09)		62 (61.166–62.834)		19 (18.862–19.138)	
Sex		< 0.001		< 0.001		< 0.001
Male	13 (12.884–13.116)		57 (55.873–58.127)		19 (18.836–19.164)	
Female	12 (11.87–12.13)		67 (65.767–68.233)		20 (19.798–20.202)	
Race		< 0.001		< 0.001		< 0.001
White	12 (11.903–12.097)		63 (62.102–63.898)		20 (19.852–20.148)	
Black	12 (11.738–12.262)		44 (41.238–46.762)		16 (15.683–16.317)	
Others	14 (13.668–14.332)		65 (61.237–68.763)		19 (18.55–19.45)	
Marital status		< 0.001		< 0.001		< 0.001
Single	11 (10.763–11.237)		46 (43.552–48.448)		15 (14.712–15.288)	
Married	14 (13.878–14.122)		75 (73.746–76.254)		22 (21.812–22.188)	
Separated	13 (12.205–13.795)		64 (56.38–71.62)		20 (18.926–21.074)	
Divorced	12 (11.7–12.3)		53 (49.791–56.209)		17 (16.624–17.376)	
Widowed	9 (8.838–9.162)		50 (48.75–51.25)		17 (16.733–17.267)	
Primary site		< 0.001		< 0.001		< 0.001
Esophagus	8 (7.818–8.182)		12 (10.137–13.863)		9 (8.819–9.181)	
Gastric	7 (6.868–7.132)		22 (19.695–24.305)		8 (7.856–8.144)	
Small intestine	9 (8.434–9.566)		28 (19.026–36.974)		9 (8.423–9.577)	
Colorectal	19 (18.856–19.144)		68 (67.137–68.863)		30 (29.793–30.207)	
Anal	18 (15.471–20.529)		20 (11.915–28.085)		19 (16.841–21.159)	
Hepato-biliary	7 (6.766–7.234)		15 (11.85–18.15)		8 (7.767–8.233)	
Pancreatic	5 (4.898–5.102)		3 (2.193–3.807)		5 (4.899–5.101)	
GIT NOS	2 (1.582–2.418)		-		1 (0.729–1.271)	
Grade		< 0.001		< 0.001		< 0.001
I	15 (14.636–15.364)		73 (70.425–75.575)		28 (27.365–28.635)	
II	17 (16.857–17.143)		64 (63.008–64.992)		26 (25.797–26.203)	
III	8 (7.91–8.09)		48 (45.931–50.069)		10 (9.886–10.114)	
IV	7 (6.632–7.368)		35 (26.333–43.667)		8 (7.555–8.445)	
Stage		< 0.001		< 0.001		
Localized	25 (24.538–25.462)		74 (72.867–75.133)		52 (51.453–52.547)	
Regional	20 (19.82–20.18)		65 (63.735–66.265)		28 (27.756–28.244)	
Distant	7 (6.925–7.075)		6 (5.471–6.529)		7 (6.926–7.074)	
Unstaged	6 (5.773–6.227)		9 (7.869–10.131)		7 (6.755–7.245)	
Chemotherapy		< 0.001		0.063		< 0.001
No	9 (8.886–9.114)		61 (60.106–61.894)		20 (19.796–20.204)	
Yes	16 (15.872–16.128)		65 (62.717–67.283)		19 (18.849–19.151)	
Radiation therapy		< 0.001		0.007		< 0.001
No	11 (10.901–11.099)		63 (62.136–63.864)		19 (18.851–19.149)	
Beam radiation	16 (15.805–16.195)		55 (51.946–58.054)		19 (18.761–19.239)	
Other types of radiation	15 (13.808–16.192)		44 (20.04–67.96)		18 (16.72–19.28)	
Surgery		< 0.001		< 0.001		< 0.001
Yes	19 (18.869–19.131)		70 (69.171–70.829)		30 (29.803–30.197)	
No	4 (3.941–4.059)		4 (3.67–4.33)		4 (3.942–4.058)	

CI = confidence interval. GIT = gastrointestinal tract. NOS = not otherwise specified.

### 3.3. Multivariate analysis of overall mortality

The hazard of overall mortality in patients with GI adenocarcinoma was higher in patients ≥ 50 years compared with patients < 50 years (HR, 1.72; 95% CI, 1.698–1.741), black compared with white patients (HR, 1.034; 95% CI, 1.023–1.044), separated (HR, 1.058; 95% CI, 1.028–1.089) and widowed (HR, 1.342; 95% CI, 1.327–1.357) compared with single patients. The hazard was higher in patients with regional (HR, 1.582; 95% CI, 1.569–1.595), distant (HR, 4.471; 95% CI, 4.426–4.516), or unstaged disease (HR, 1.904; 95% CI, 1.872–1.938) compared with localized; and in patients with grade II (HR, 1.057; 95% CI, 1.045–1.068), III (HR, 1.36; 95% CI, 1.344–1.376), or IV (HR, 1.459; 95% CI, 1.42–1.498) compared with grade I tumor. The hazard was higher in patients not treated by surgery (HR, 2.541; 95% CI, 2.513–2.57).

The hazard of overall mortality was lower in females (HR, 0.847; 95% CI, 0.841–0.853), races other than black and white compared with white (HR, 0.842; 95% CI, 0.832–0.852), married (HR, 0.85; 95% CI, 0.841–0.859) and divorced (HR, 0.953; 95% CI, 0.94–0.967) compared with single patients, patients treated with chemotherapy compared with not (HR, 0.623; 95% CI, 0.618–0.627), patients treated with beam (HR, 0.872; 95% CI, 0.858–0.885) or other types of radiation (HR, 0.901; 95% CI, 0.857–0.946) compared with no radiation therapy.

Regarding the site of the AC; higher overall mortality hazards were associated with gastric (HR, 1.052; 95% CI, 1.033–1.07), hepatobiliary (HR, 1.445; 95% CI, 1.414–1.477), pancreatic (HR, 1.496; 95% CI, 1.468–1.523), and non-specific GI adenocarcinoma (HR, 1.328; 95% CI, 1.225–1.439) compared with esophageal AC. Lower hazards were associated with colorectal (HR, 0.649; 95% CI, 0.639–0.66) and anal AC (HR, 0.802; 95% CI, 0.728–0.882) compared with esophageal AC. Small intestinal AC had similar hazard as esophageal AC (HR, 1.01; 95% CI, 0.976–1.046) (Table 3).

**Table 3:** Multivariate analysis using Cox regression test.

Variables	Total mortality, HR (95% CI)	Regression coefficient	Adenocarcinoma mortality, HR (95% CI)	Regression coefficient	Cardiovascular mortality, HR (95% CI)	Regression coefficient
Age, reference (< 50 years)						
≥ 50 years	1.72 (1.698–1.741)^b^	0.542	1.245 (1.228–1.262)^b^	0.219	8.476 (7.91–9.083)^b^	2.137
Sex, reference (male)						
Female	0.847 (0.841–0.853)^b^	-0.166	0.916 (0.908–0.924)^b^	-0.088	0.729 (0.717–0.742)^b^	-0.316
Race, reference (white)						
Black	1.034 (1.023–1.044)^b^	0.033	1.074 (1.061–1.088)^b^	0.072	0.95 (0.924–0.978)^b^	-0.051
Others	0.842 (0.832–0.852)^b^	-0.172	0.907 (0.894–0.92)^b^	-0.097	0.708 (0.685–0.732)^b^	-0.345
Marital status, reference (single)						
Married	0.85 (0.841–0.859)^b^	-0.163	0.895 (0.884–0.906)^b^	-0.111	0.77 (0.749–0.792)^b^	-0.261
Separated	1.058 (1.028–1.089)^b^	0.056	1.016 (0.98–1.053)	0.016	1.27 (1.184–1.361)^b^	0.239
Divorced	0.953 (0.94–0.967)^b^	-0.048	0.974 (0.957–0.991)^a^	-0.027	0.841 (0.807–0.877)^b^	-0.173
Widowed	1.342 (1.327–1.357)^b^	0.294	1.189 (1.172–1.206)^b^	0.173	1.867 (1.812–1.924)^b^	0.624
Primary site, reference (esophagus)						
gastric	1.052 (1.033–1.07)^b^	0.05	1.068 (1.048–1.089)^b^	0.066	1.18 (1.1–1.265)^b^	0.165
small intestine	1.01 (0.976–1.046)	0.01	1.001 (0.962–1.041)	0.001	1.019 (0.898–1.157)	0.019
colorectal	0.649 (0.639–0.66)^b^	-0.432	0.568 (0.557–0.578)^b^	-0.566	1.123 (1.053–1.198)^b^	0.116
anal	0.802 (0.728–0.882)^b^	-0.221	0.776 (0.693–0.868)^b^	-0.254	1.196 (0.9–1.588)	0.179
hepatobiliary	1.445 (1.414–1.477)^b^	0.368	1.509 (1.473–1.546)^b^	0.411	1.017 (0.931–1.111)	0.017
pancreatic	1.496 (1.468–1.523)^b^	0.402	1.553 (1.522–1.584)^b^	0.44	0.83 (0.757–0.91)^b^	-0.186
GIT NOS	1.328 (1.225–1.439)^b^	0.283	0.763 (0.684–0.852)^b^	-0.27	1.096 (0.695–1.726)	0.091
Stage, reference (localized)						
Regional	1.582 (1.569–1.595)^b^	0.459	2.454 (2.424–2.485)^b^	0.898	1.124 (1.105–1.143)^b^	0.117
Distant	4.471 (4.426–4.516)^b^	1.498	8.166 (8.056–8.278)^b^	2.1	1.064 (1.026–1.102)^b^	0.062
Unstaged	1.904 (1.872–1.938)^b^	0.644	3.159 (3.091–3.228)^b^	1.15	1.439 (1.372–1.509)^b^	0.364
Grade, reference (grade I)						
II	1.057 (1.045–1.068)^b^	0.055	1.092 (1.076–1.108)^b^	0.088	0.966 (0.943–0.989)^a^	-0.035
III	1.36 (1.344–1.376)^b^	0.308	1.497 (1.474–1.52)^b^	0.403	1.058 (1.028–1.089)^b^	0.057
IV	1.459 (1.42–1.498)^b^	0.378	1.607 (1.556–1.658)^b^	0.474	1.157 (1.068–1.255)^b^	0.146
Chemotherapy, reference (no/unknown)						
Yes	0.623 (0.618–0.627)^b^	-0.474	0.663 (0.657–0.669)^b^	-0.412	0.416 (0.406–0.427)^b^	-0.876
Radiation therapy, reference (no radiation)						
Beam radiation	0.872 (0.858–0.885)^b^	-0.137	0.887 (0.872–0.902)^b^	-0.12	0.942 (0.881–1.006)	-0.06
Other types of radiation	0.901 (0.857–0.946)^b^	-0.105	0.929 (0.879–0.981)^a^	-0.074	0.844 (0.693–1.028)	-0.169
Surgery, reference (surgery performed)						
No	2.541 (2.513–2.57)^b^	0.933	2.507 (2.476–2.538)^b^	0.919	2.04 (1.958–2.126)^b^	0.713

CI = confidence interval. HR = hazard ratio. GIT = gastrointestinal tract. NOS = not otherwise specified. ^a^P < 0.01. ^b^P < 0.001.

### 3.4. Multivariate analysis of cardiovascular specific mortality

The hazard of CV mortality in GI adenocarcinoma patients was higher in patients ≥ 50 years compared with patients < 50 years (HR, 8.476; 95% CI, 7.91–9.083), separated (HR, 1.27; 95% CI, 1.184–1.361) and widowed (HR, 1.867; 95% CI, 1.812–1.924) compared with single patients, patients with regional (HR, 1.124; 95% CI, 1.105–1.143), distant (HR, 1.064; 95% CI, 1.026–1.102) or unstaged disease (HR, 1.439; 95% CI, 1.372–1.509) compared with localized stage, patients with grade III (HR, 1.058; 95% CI, 1.028–1.089) or IV (HR, 1.157; 95% CI, 1.068–1.255) tumor compared with grade I, and in patients not treated with surgery compared with patients treated with surgery (HR, 2.04; 95% CI, 1.958–2.126).

The hazard of CV mortality was lower in females (HR, 0.729; 95% CI, 0.717–0.742), black (HR, 0.95; 95% CI, 0.924–0.978) and other races (HR, 0.708; 95% CI, 0.685–0.732) compared with white race, married (HR, 0.77; 95% CI, 0.749–0.792) and divorced (HR, 0.841; 95% CI, 0.807–0.877) compared with single patients, patients with grade II of the disease compared with grade I (HR, 0.966; 95% CI, 0.943–0.989), and in patients treated with chemotherapy compared with patients not treated with chemotherapy (HR, 0.416; 95% CI, 0.406–0.427). Treatment with beam or other types of radiation had no significant effect on CV mortality.

Regarding the site of AC, gastric (HR, 1.18; 95% CI, 1.1–1.265) and colorectal AC (HR, 1.123; 95% CI, 1.053–1.198) had higher hazards of CV mortality, while pancreatic AC had lower hazard (HR, 0.83; 95% CI, 0.757–0.91) compared with esophageal AC. Hazards in small intestinal (HR, 1.019; 95% CI, 0.898–1.157), anal (HR, 1.196; 95% CI, 0.9–1.588), hepatobiliary (HR, 1.017; 95% CI, 0.931–1.111), and non-specific GI adenocarcinoma (HR, 1.096; 95% CI, 0.695–1.726) were similar to that of the esophageal AC (Table 3).

### 3.5. Multivariate analysis of adenocarcinoma-specific mortality

The hazard for adenocarcinoma-specific mortality was higher in patients ≥ 50 years compared with younger patients, black compared with white patients, widowed compared with single patients, patients with regional, distant or unstaged disease compared with localized stage, patients with grade II, III or IV compared with grade I tumor, and patients not treated with cancer directed surgery. The hazard was lower in females, races other than black and white compared with white, married and divorced compared with single patients, patients treated with chemotherapy compared with not, and in patients treated with beam or other types of radiation compared with no radiation. Separated patients had similar hazard as single. Regarding the site of the AC; higher AC-mortality hazard was associated with gastric, hepatobiliary and pancreatic AC, while lower AC-mortality hazard was associated with colorectal, anal and non-specific GI adenocarcinoma compared with esophageal AC. Small intestinal AC had similar hazard as esophageal AC (Table 3).

### 3.6. Binary logistic regression of cardiovascular mortality

Among patients with GI adenocarcinoma, higher probability of CV disease was associated with age ≥ 50 years compared with age < 50 years (OR, 5.917; 95% CI, 5.518–6.345); married (OR, 1.054; 95% CI, 1.022–1.087), separated (OR, 1.465; 95% CI, 1.356–1.582), and widowed (OR, 0.904; 95% CI, 0.864–0.945) compared with single patients; and beam radiation therapy compared with no radiation (OR, 1.204; 95% CI, 1.123–1.291). Lower probability of CV disease was associated with female sex (OR, 0.857; 95% CI, 0.841–0.873), black (OR, 0.872; 95% CI, 0.845–0.899) and other races (OR, 0.709; 95% CI, 0.685–0.735) compared with white, divorced status compared with single (OR, 0.904; 95% CI, 0.864–0.945), regional (OR, 0.854; 95% CI, 0.838–0.871) and distant (OR, 0.27; 95% CI, 0.261–0.279) stages compared with localized disease, grade II (OR, 0.787; 95% CI, 0.767–0.809), III (OR, 0.75; 95% CI, 0.727–0.775) or IV (OR, 0.635; 95% CI, 0.582–0.692) compared with grade I tumor, chemotherapy compared with no or unknown history of chemotherapy (OR, 0.411; 95% CI, 0.401–0.422), and no surgery compared with cancer directed surgery (OR, 0.689; 95% CI, 0.661–0.718). Types of radiation other than beam had no significant effect on the probability of CV disease (Table 4).

**Table 4:** The probability of cardiovascular disease using binary logistic regression test.

Variables	Cardiovascular, OR (95% CI)	Regression coefficient
Age, reference (< 50 years)		
≥ 50 years	5.917 (5.518–6.345)^b^	2.137
Sex, reference (male)		
Female	0.857 (0.841–0.873)^b^	-0.316
Race, reference (white)		
Black	0.872 (0.845–0.899)^b^	-0.051
Others	0.709 (0.685–0.735)^b^	-0.345
Marital status, reference (single)		
Married	1.054 (1.022–1.087)^b^	-0.261
Separated	1.465 (1.356–1.582)^b^	0.239
Divorced	0.904 (0.864–0.945)^b^	-0.173
Widowed	1.777 (1.72–1.836)^b^	0.624
Primary site, reference (esophagus)		
gastric	1.256 (1.168–1.352)^b^	0.165
small intestine	1.042 (0.912–1.191)	0.019
colorectal	1.928 (1.804–2.061)^b^	0.116
anal	1.453 (1.071–1.972)^a^	0.179
hepatobiliary	0.695 (0.634–0.762)^b^	0.017
pancreatic	0.562 (0.512–0.618)^b^	-0.186
GIT NOS	0.927 (0.593–1.449)	0.091
Stage, reference (localized)		
Regional	0.854 (0.838–0.871)^b^	0.117
Distant	0.27 (0.261–0.279)^b^	0.062
Unstaged	1.014 (0.963–1.067)	0.364
Grade, reference (grade I)		
II	0.787 (0.767–0.809)^b^	-0.035
III	0.75 (0.727–0.775)^b^	0.057
IV	0.635 (0.582–0.692)^b^	0.146
Chemotherapy, reference (no/unknown)		
Yes	0.411 (0.401–0.422)^b^	-0.876
Radiation therapy, reference (no radiation)		
Beam radiation	1.204 (1.123–1.291)^b^	-0.06
Other types of radiation	1.008 (0.819–1.241)	-0.169
Surgery, reference (surgery performed)		
No	0.689 (0.661–0.718)^b^	0.713

CI = confidence interval. OR = odds ratio. GIT = gastrointestinal tract. NOS = not otherwise specified. ^a^P < 0.05. ^b^P < 0.001.

Regarding the site of the AC; higher CV disease probability was associated gastric (OR, 1.256; 95% CI, 1.168–1.352), colorectal (OR, 1.928; 95% CI, 1.804–2.061), and anal AC (OR, 1.453; 95% CI, 1.071–1.972), while lower probability was associated with hepatobiliary (OR, 0.695; 95% CI, 0.634–0.762) and pancreatic AC (OR, 0.562; 95% CI, 0.512–0.618) compared with esophageal AC. Small intestinal (OR, 1.042; 95% CI, 0.912–1.191) and non-specific GI adenocarcinoma (OR, 0.927; 95% CI, 0.593–1.449) had similar probability as esophageal AC (Table 4).

## 4. Discussion

We analyzed data of 556,350 patients with adenocarcinoma at any site of the GI tract and its accessory glands. We found that gastric and colorectal adenocarcinomas (ACs) have higher CV mortality rates, while pancreatic AC has a lower CV mortality rate compared with esophageal AC. Anal, small intestinal and hepatobiliary ACs have CV mortality rates similar to that of the esophageal AC. Independent patients’ characteristics associated with higher CV mortality rates include older age at diagnosis (≥ 50 years), male sex, white race, separated and widowed statuses, non-localized disease stages (regional, distant and unstaged disease), grades III and IV of the tumor, no or unknown history of chemotherapy and no history of cancer-directed surgery. Protective factors include married and divorced statuses, and grade II of the disease. Radiation therapy has no predictive value for CV mortality.

Our results revealed that older age, male sex, and no cancer-directed surgery are independent risk factors for CV mortality in GI adenocarcinoma patients. These findings agree with the findings of a previous study on CRC patients [27]. This may be due to the large doses of cardiotoxic cancer therapies that may have been used in patients not undergone surgery. The same study identified black race as an independent risk factor for CV mortality in patients with CRC [27], but our results showed that it is a protective factor from CV mortality. This may be because they included patients registered between 2010 and 2014 only, which indicates a short period of follow-up in their study [27]. Another study found that older age, male sex, black race, chemotherapy, advanced grade, and advanced stage are risk factors for CRC-specific mortality [28]. For non-cancer mortality, the same study identified older age, male sex, advanced grade and advanced stage as risk factors while chemotherapy was protective [28]. A previous study on gall bladder adenocarcinoma patients stated that older age, black race, and advanced grade of the tumor are risk factors for adenocarcinoma mortality; and that older age, advanced grade of the tumor, and chemotherapy are risk factors for non-cancer mortality [29]. Cardiovascular deaths were the commonest among non-cancer deaths in patients with CRC in a previous SEER based study, which agrees with our findings [30].

Previous studies stated that CV mortality is higher in cancer patients compared with the general population [31–34]. This finding was also reported in patients with CRC [27, 35], breast cancer [36, 37], and in children and young cancer patients [17, 38]. The risk of non-cancer mortality remained high in cancer patients for many years after diagnosis [17]. The increased CV mortality in these patients may be due to the use of cardiotoxic cancer-directed therapies, which may lead to impaired CV function and increase the CV mortality risk [39, 40].

For total mortality in GI adenocarcinoma patients, risk factors included older age, male sex, black race, separated and widowed statuses, non-localized disease stages (regional, distant, unstaged), higher disease grades (grades II, III, IV), no or unknown history of chemotherapy, no radiation therapy and no history of cancer-directed surgery. Protective factors included races other than black and white, married and divorced statuses, chemotherapy, radiotherapy and surgery. Regarding the site of adenocarcinoma; gastric, hepatobiliary and pancreatic adenocarcinoma had higher mortality rates, while colorectal and anal adenocarcinomas had lower mortalities compared with the esophageal AC. The mortality rate in small intestinal AC was similar to that of the esophagus.

Previous studies on patients with GI adenocarcinomas (including colorectal, small intestinal and pancreatic ACs) agree that advanced disease stage (including lymph node invasion and distant spread) and advanced tumor grade are risk factors for overall mortality [41–44]. Patient-related risk factors included advanced age, unmarried status, and non-white races [41–45]. Surgical treatment was found to be a protective factor in small intestinal and pancreatic ACs [42–44].

A study on pancreatic AC stated that male sex is another risk factor [45], but a study on small intestinal AC reported that sex has no predictive value for mortality [43]. This may be due to the relatively small number of small intestinal AC patients included in that study [43]. The same study stated that small intestinal AC has a worse prognosis than colorectal AC, which coincides with our results [43].

For adenocarcinoma-specific mortality, risk factors included older age, male sex, black race, widowed state, non-localized stages of the disease (regional, distant and unstaged), advanced grade (grades II, III and IV), no chemotherapy, no radiotherapy and no surgery performed. Protective factors included races other than black and white, and married and divorced statuses. Gastric, hepatobiliary, and pancreatic adenocarcinomas had higher mortalities, while colorectal and anal carcinomas had lower mortalities than esophageal and small intestinal adenocarcinomas.

Previous SEER-based studies on small intestinal AC agree that older age, advanced disease stage and grade, and unmarried status are risk factors for cancer-specific mortality, while performed surgery is protective [44, 46]. A study that included Chinese patients with small intestinal AC found that lymph node invasion was the only significant risk factors for cancer-specific mortality in this group [46].

This study has the strengths of the large sample size and the high-quality data. These factors help identification of the independent risk factors and generalization of the results. However, it is limited to the retrospective study design and the lack of some data that may have affected the cardiovascular health and disease, for example, the family history, dyslipidemia and diabetes. It also depends on data from different health care facilities that may have different levels of care.

## 5. Conclusion

Among patients with GI adenocarcinoma; those with higher risk for CV mortality include older patients, males, white, separated and widowed, patients with gastric or colorectal adenocarcinoma, patients with advanced grade or advanced stage of the disease, patients not treated with chemotherapy and those not undergone cancer-directed surgery. The lower risk was found in married, divorced patients, and patients with pancreatic adenocarcinoma.

## Supporting information

S1 File Survival curves. The file shows survival curves for cancer-Specific and cardiovascular mortalities with different independent variables (age, sex, marital status, race, cancer type, site, grade, stage, chemotherapy, radiation therapy, and surgery).
(DOCX)

## References

[1] Rawla P, Barsouk A: Epidemiology of gastric cancer: global trends, risk factors and prevention. Przeglad gastroenterologiczny. 2019, 14:26–38. doi: 10.5114/pg.2018.80001 30944675 PMC6444111

[2] Allemani C, Matsuda T, Di Carlo V, Harewood R, Matz M, Niksic M, et al: Global surveillance of trends in cancer survival 2000–14 (CONCORD-3): analysis of individual records for 37 513 025 patients diagnosed with one of 18 cancers from 322 population-based registries in 71 countries. Lancet (London, England). 2018, 391:1023–1075. doi: 10.1016/s0140-6736(17)33326-3 29395269 PMC5879496

[3] Arnold M, Rutherford MJ, Bardot A, Ferlay J, Andersson TM, Myklebust TA, et al: Progress in cancer survival, mortality, and incidence in seven high- income countries 1995–2014 (ICBP SURVMARK-2): a population-based study. The Lancet Oncology. 2019, 20:1493–1505. doi: 10.1016/S1470-2045(19)30456-5 31521509 PMC6838671

[4] Sung H, Ferlay J, Siegel RL, Laversanne M, Soerjomataram I, Jemal A, Bray F: Global cancer statistics 2020: GLOBOCAN estimates of incidence and mortality worldwide for 36 cancers in 185 countries. CA: a cancer journal for clinicians. 2021. doi: 10.3322/caac.21660 33538338

[5] Fidler MM, Soerjomataram I, Bray F: A global view on cancer incidence and national levels of the human development index. International journal of cancer. 2016, 139:2436–2446. doi: 10.1002/ijc.30382 27522007

[6] Johnson CM, Wei C, Ensor JE, Smolenski DJ, Amos CI, Levin B, et al: Meta-analyses of colorectal cancer risk factors. Cancer causes & control: CCC. 2013, 24:1207–1222. doi: 10.1007/s10552-013-0201-5 23563998 PMC4161278

[7] Kuipers EJ, Grady WM, Lieberman D, Seufferlein T, Sung JJ, Boelens PG, et al: Colorectal cancer. Nature reviews Disease primers. 2015, 1:15065. doi: 10.1038/nrdp.2015.65 27189416 PMC4874655

[8] Smyth EC, Verheij M, Allum W, Cunningham D, Cervantes A, Arnold D: Gastric cancer: ESMO clinical practice guidelines for diagnosis, treatment and follow-up. Annals of oncology: official journal of the European Society for Medical Oncology. 2016, 27:v38–v49. doi: 10.1093/annonc/mdw350 27664260

[9] Plummer M, Franceschi S, Vignat J, Forman D, de Martel C: Global burden of gastric cancer attributable to Helicobacter pylori. International journal of cancer. 2015, 136:487–490. doi: 10.1002/ijc.28999 24889903

[10] McGlynn KA, Petrick JL, London WT: Global epidemiology of hepatocellular carcinoma: an emphasis on demographic and regional variability. Clinics in liver disease. 2015, 19:223–238. doi: 10.1016/j.cld.2015.01.001 25921660 PMC4712629

[11] Vogel A, Cervantes A, Chau I, Daniele B, Llovet JM, Meyer T, et al: Hepatocellular carcinoma: ESMO Clinical Practice Guidelines for diagnosis, treatment and follow-up. Annals of oncology: official journal of the European Society for Medical Oncology. 2018, 29:iv238–iv255. doi: 10.1093/annonc/mdy308 30285213

[12] Marengo A, Rosso C, Bugianesi E: Liver Cancer: Connections with Obesity, Fatty Liver, and Cirrhosis. Annual review of medicine. 2016, 67:103–117. doi: 10.1146/annurev-med-090514-013832 26473416

[13] Harris C, Croce B, Munkholm-Larsen S: Esophageal cancer. Annals of cardiothoracic surgery. 2017, 6:190. doi: 10.21037/acs.2017.03.01 28447010 PMC5387150

[14] Lordick F, Mariette C, Haustermans K, Obermannova R, Arnold D: Oesophageal cancer: ESMO clinical practice guidelines for diagnosis, treatment and follow-up. Annals of oncology: official journal of the European Society for Medical Oncology. 2016, 27:v50–v57. doi: 10.1093/annonc/mdw329 27664261

[15] Edwards BK, Noone AM, Mariotto AB, Simard EP, Boscoe FP, Henley SJ, et al: Annual Report to the Nation on the status of cancer, 1975–2010, featuring prevalence of comorbidity and impact on survival among persons with lung, colorectal, breast, or prostate cancer. Cancer. 2014, 120:1290–1314. doi: 10.1002/cncr.28509 24343171 PMC3999205

[16] Keegan TH, Ries LA, Barr RD, Geiger AM, Dahlke DV, Pollock BH, et al: Comparison of cancer survival trends in the United States of adolescents and young adults with those in children and older adults. Cancer. 2016, 122:1009–1016. doi: 10.1002/cncr.29869 26848927

[17] Anderson C, Lund JL, Weaver MA, Wood WA, Olshan AF, Nichols HB: Noncancer mortality among adolescents and young adults with cancer. Cancer. 2019, 125:2107–2114. doi: 10.1002/cncr.32063 30892701 PMC7001100

[18] Coccia PF, Pappo AS, Beaupin L, Borges VF, Borinstein SC, Chugh R, et al: Adolescent and Young Adult Oncology, Version 2.2018, NCCN Clinical Practice Guidelines in Oncology. Journal of the National Comprehensive Cancer Network: JNCCN. 2018, 16:66–97. doi: 10.6004/jnccn.2018.0001 29295883

[19] Chen CL: Cardiovascular prevention in the cancer survivor. Current atherosclerosis reports. 2015, 17:484. doi: 10.1007/s11883-014-0484-3 25637040

[20] Douglas PS, Garcia MJ, Haines DE, Lai WW, Manning WJ, Patel AR, et al: ACCF/ASE/AHA/ASNC/HFSA/HRS/SCAI/SCCM/SCCT/SCMR 2011 appropriate use criteria for echocardiography. Journal of the American Society of Echocardiography: official publication of the American Society of Echocardiography. 2011, 24:229–267. doi: 10.1016/j.echo.2010.12.008 21338862

[21] Curigliano G, Cardinale D, Dent S, Criscitiello C, Aseyev O, Lenihan D, et al: Cardiotoxicity of anticancer treatments: Epidemiology, detection, and management. CA: a cancer journal for clinicians. 2016, 66:309–325. doi: 10.3322/caac.21341 26919165

[22] Lenneman CG, Sawyer DB: Cardio-Oncology: An Update on Cardiotoxicity of Cancer-Related Treatment. Circulation research. 2016, 118:1008–1020. doi: 10.1161/CIRCRESAHA.115.303633 26987914

[23] Steinherz LJ, Steinherz PG, Tan CT, Heller G, Murphy ML: Cardiac toxicity 4 to 20 years after completing anthracycline therapy. Jama. 1991, 266:1672–1677. 1886191

[24] McHugh ML: The chi-square test of independence. Biochem Med (Zagreb). 2013, 23:143–149. doi: 10.11613/bm.2013.018 23894860 PMC3900058

[25] Clark TG, Bradburn MJ, Love SB, Altman DG: Survival analysis part I: basic concepts and first analyses. Br J Cancer. 2003, 89:232–238. doi: 10.1038/sj.bjc.6601118 12865907 PMC2394262

[26] Nick TG, Campbell KM: Logistic Regression. Topics in Biostatistics. Ambrosius WT(ed): Humana Press, Totowa, NJ; 2007:273–301. doi: 10.1007/978-1-59745-530-5_14

[27] Gaitanidis A, Spathakis M, Tsalikidis C, Alevizakos M, Tsaroucha A, Pitiakoudis M: Risk factors for cardiovascular mortality in patients with colorectal cancer: a population-based study. International journal of clinical oncology. 2019, 24:501–507. doi: 10.1007/s10147-018-01382-x 30604158

[28] Wang R, Han L, Dai W, Mo S, Xiang W, Li Q, et al: Cause of death for elders with colorectal cancer: a real-world data analysis. Journal of gastrointestinal oncology. 2020, 11:269–276. doi: 10.21037/jgo.2020.03.04 32399268 PMC7212099

[29] Han D, Yang J, Xu F, Huang Q, Bai L, Wei YL, et al: Prognostic factors in patients with gallbladder adenocarcinoma identified using competing-risks analysis: A study of cases in the SEER database. Medicine. 2020, 99:e21322. doi: 10.1097/MD.0000000000021322 32756116 PMC7402769

[30] Chen J, Zheng Y, Wang H, Zhang D, Zhao L, Yu D, et al: Cause of death among patients with colorectal cancer: a population-based study in the United States. Aging. 2020, 12:22927–22948. doi: 10.18632/aging.104022 33289707 PMC7746372

[31] Henson KE, Reulen RC, Winter DL, Bright CJ, Fidler MM, Frobisher C, et al: Cardiac Mortality Among 200 000 Five-Year Survivors of Cancer Diagnosed at 15 to 39 Years of Age: The Teenage and Young Adult Cancer Survivor Study. Circulation. 2016, 134:1519–1531. doi: 10.1161/CIRCULATIONAHA.116.022514 27821538 PMC5106083

[32] Barac A, Murtagh G, Carver JR, Chen MH, Freeman AM, Herrmann J, et al: Cardiovascular Health of Patients With Cancer and Cancer Survivors: A Roadmap to the Next Level. Journal of the American College of Cardiology. 2015, 65:2739–2746. doi: 10.1016/j.jacc.2015.04.059 26112199 PMC4484773

[33] Saiki H, Petersen IA, Scott CG, Bailey KR, Dunlay SM, Finley RR, et al: Risk of Heart Failure With Preserved Ejection Fraction in Older Women After Contemporary Radiotherapy for Breast Cancer. Circulation. 2017, 135:1388–1396. doi: 10.1161/CIRCULATIONAHA.116.025434 28132957 PMC5388583

[34] Thavendiranathan P, Abdel-Qadir H, Fischer HD, Camacho X, Amir E, Austin PC, et al: Breast Cancer Therapy-Related Cardiac Dysfunction in Adult Women Treated in Routine Clinical Practice: A Population-Based Cohort Study. Journal of clinical oncology: official journal of the American Society of Clinical Oncology. 2016, 34:2239–2246. doi: 10.1200/JCO.2015.65.1505 27091709

[35] Weaver KE, Foraker RE, Alfano CM, Rowland JH, Arora NK, Bellizzi KM, et al: Cardiovascular risk factors among long-term survivors of breast, prostate, colorectal, and gynecologic cancers: a gap in survivorship care? Journal of cancer survivorship: research and practice. 2013, 7:253–261. doi: 10.1007/s11764-013-0267-9 23417882 PMC3756807

[36] Effects of chemotherapy and hormonal therapy for early breast cancer on recurrence and 15-year survival: an overview of the randomised trials. Lancet (London, England). 2005, 365:1687–1717. doi: 10.1016/S0140-6736(05)66544-0 15894097

[37] Clarke M, Collins R, Darby S, Davies C, Elphinstone P, Evans V, et al: Effects of radiotherapy and of differences in the extent of surgery for early breast cancer on local recurrence and 15-year survival: an overview of the randomised trials. Lancet (London, England). 2005, 366:2087–2106. doi: 10.1016/S0140-6736(05)67887-7 16360786

[38] Mertens AC, Liu Q, Neglia JP, Wasilewski K, Leisenring W, Armstrong GT, et al: Cause-specific late mortality among 5-year survivors of childhood cancer: the Childhood Cancer Survivor Study. Journal of the National Cancer Institute. 2008, 100:1368–1379. doi: 10.1093/jnci/djn310 18812549 PMC2556702

[39] Herrmann J, Lerman A, Sandhu NP, Villarraga HR, Mulvagh SL, Kohli M: Evaluation and management of patients with heart disease and cancer: cardio-oncology. Mayo Clinic proceedings. 2014, 89:1287–1306. doi: 10.1016/j.mayocp.2014.05.013 25192616 PMC4258909

[40] Kapelakis I, Toutouzas K, Drakopoulou M, Michelongona A, Zagouri F, Mpamias A, et al: Bevacizumab increases the incidence of cardiovascular events in patients with metastatic breast or colorectal cancer. Hellenic journal of cardiology: HJC = Hellenike kardiologike epitheorese. 2017, 58:215–219. doi: 10.1016/j.hjc.2016.11.022 28258825

[41] Emre A, Akbulut S, Sertkaya M, Bitiren M, Kale IT, Bulbuloglu E: Assessment of risk factors affecting mortality in patients with colorectal cancer. Przeglad gastroenterologiczny. 2018, 13:109–117. doi: 10.5114/pg.2018.73348 30002769 PMC6040099

[42] Salami A, Obaid T, Joshi ART: Trends in the clinical presentation, treatment, and survival for pancreatic adenocarcinoma. American journal of surgery. 2019, 217:103–107. doi: 10.1016/j.amjsurg.2018.05.017 29807632

[43] Young JI, Mongoue-Tchokote S, Wieghard N, Mori M, Vaccaro GM, Sheppard BC, et al: Treatment and Survival of Small-bowel Adenocarcinoma in the United States: A Comparison With Colon Cancer. Diseases of the colon and rectum. 2016, 59:306–315. doi: 10.1097/DCR.0000000000000562 26953989

[44] Zheng Z, Zhou X, Zhang J, Zhao B, Chen C, Liu X, et al: Nomograms predict survival of patients with small bowel adenocarcinoma: a SEER-based study. International journal of clinical oncology. 2021, 26:387–398. doi: 10.1007/s10147-020-01813-8 33113018

[45] Saad AM, Turk T, Al-Husseini MJ, Abdel-Rahman O: Trends in pancreatic adenocarcinoma incidence and mortality in the United States in the last four decades; a SEER-based study. BMC cancer. 2018, 18:688. doi: 10.1186/s12885-018-4610-4 29940910 PMC6020186

[46] Jiang S, Zhao R, Li Y, Han X, Liu Z, Ge W, et al: Prognosis and nomogram for predicting postoperative survival of duodenal adenocarcinoma: A retrospective study in China and the SEER database. Scientific reports. 2018, 8:7940. doi: 10.1038/s41598-018-26145-6 29786691 PMC5962558

