# Supplementary material for: Incidence and risk factors of cardiovascular mortality in patients with gastrointestinal adenocarcinoma
Source: PLoS One. 2023 Jan 27;18(1):e0262013. doi: 10.1371/journal.pone.0262013 (PMC9882755; doi:10.1371/journal.pone.0262013)
Supplement: S1 File — The file shows survival curves for cancer-Specific and cardiovascular mortalities with different independent variables (age, sex, marital status, race, cancer type, site, grade, stage, chemotherapy, radiation therapy, and surgery). (DOCX) [file pone.0262013.s001.docx]

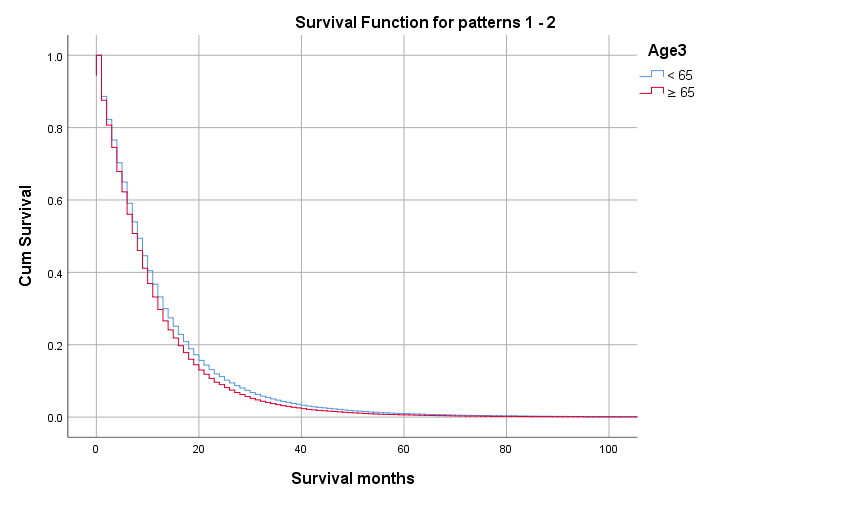


Fig.S1 Survival Curve for Cancer-Specific Mortality with Age.


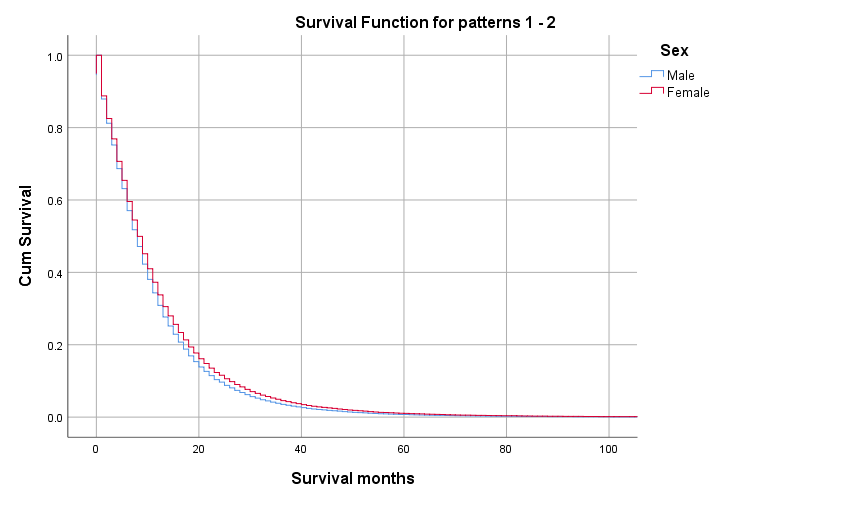


Fig.S2 Survival Curve for Cancer-Specific Mortality with Sex.


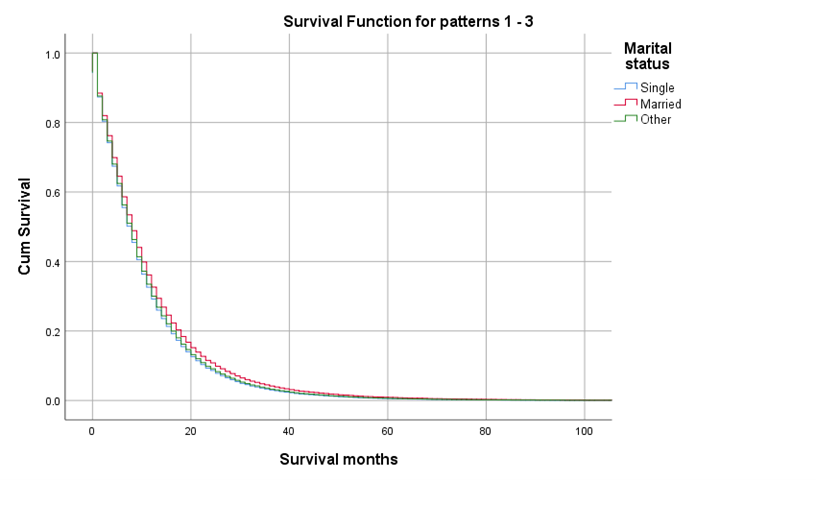


Fig.S3 Survival Curve for Cancer-Specific Mortality with Marital status.


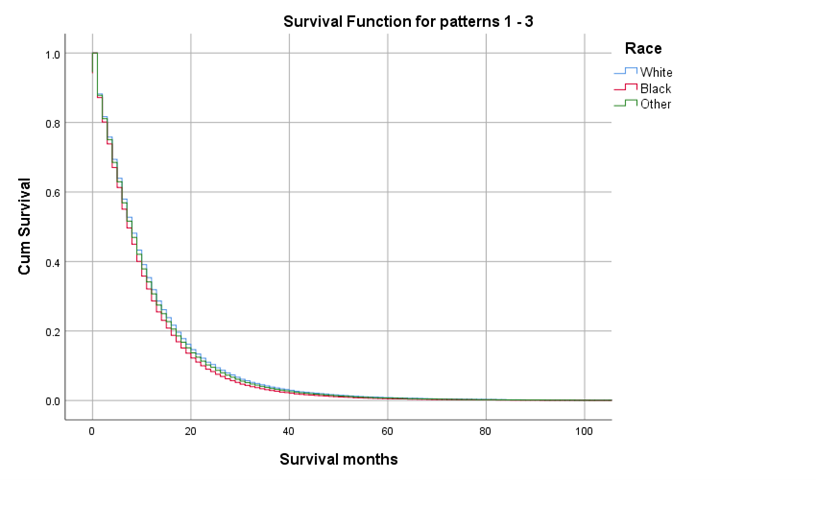


Fig.S4 Survival Curve for Cancer-Specific Mortality with Race.


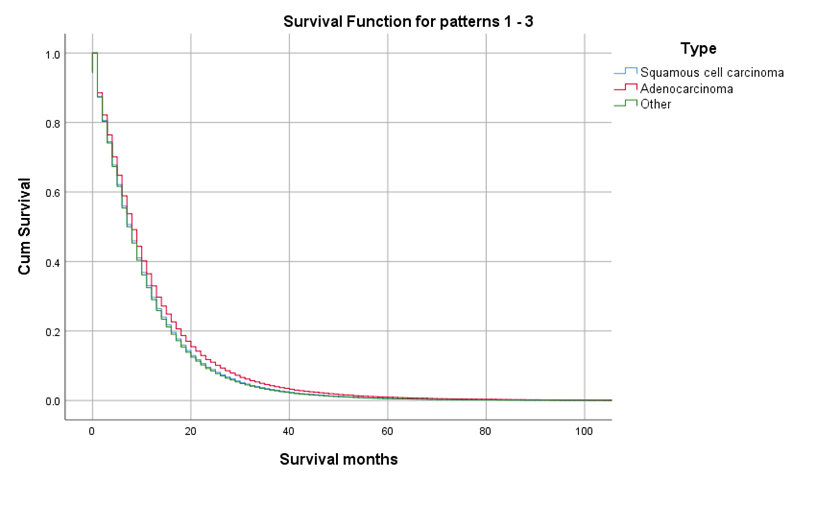


Fig.S5 Survival Curve for Cancer-Specific Mortality with Type.


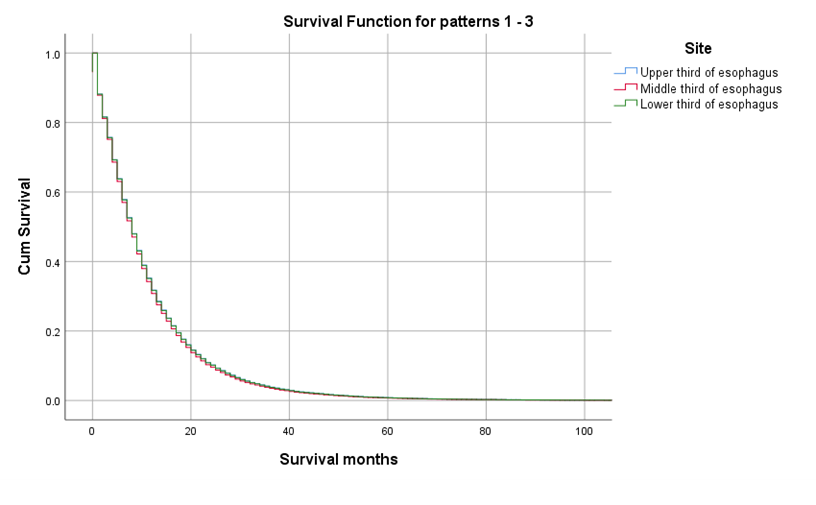


Fig.S6 Survival Curve for Cancer-Specific Mortality with Site.


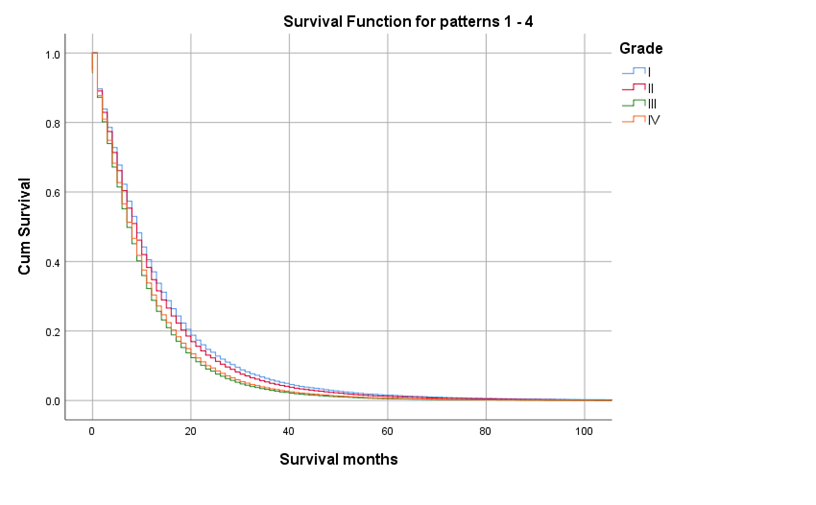


Fig.S7 Survival Curve for Cancer-Specific Mortality with Grade.


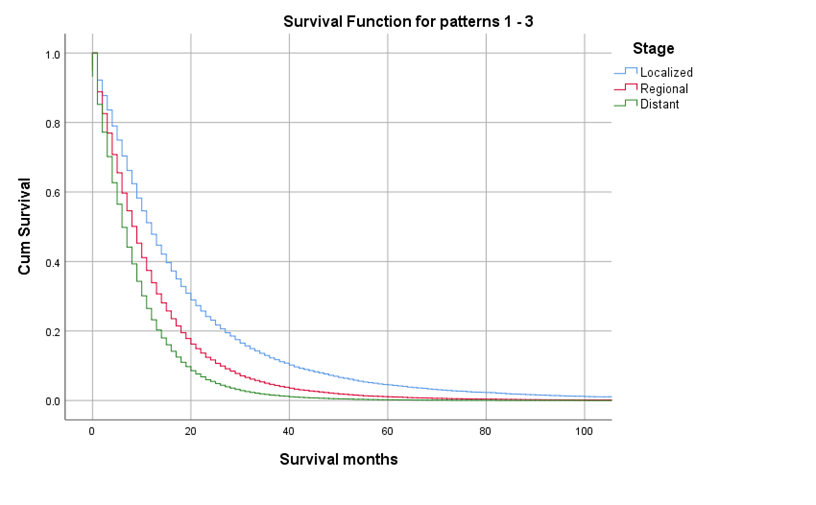


Fig.S8 Survival Curve for Cancer-Specific Mortality with Stage.


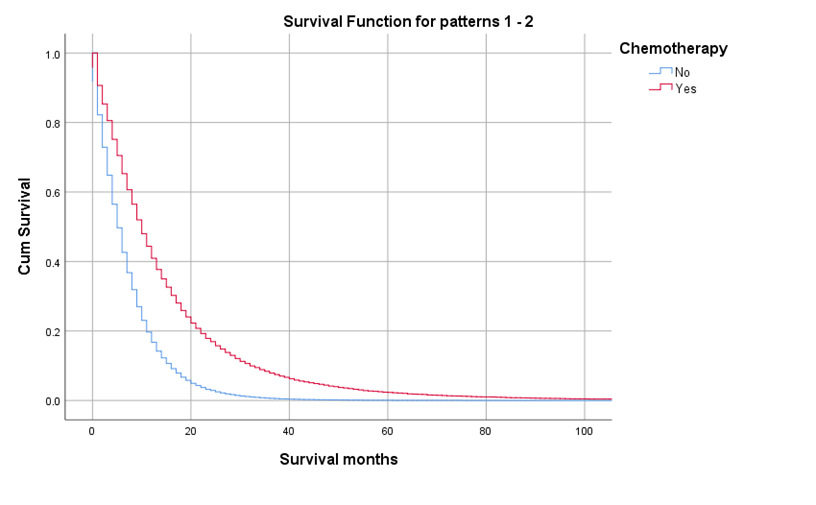


Fig.S9 Survival Curve for Cancer-Specific Mortality with Chemotherapy.


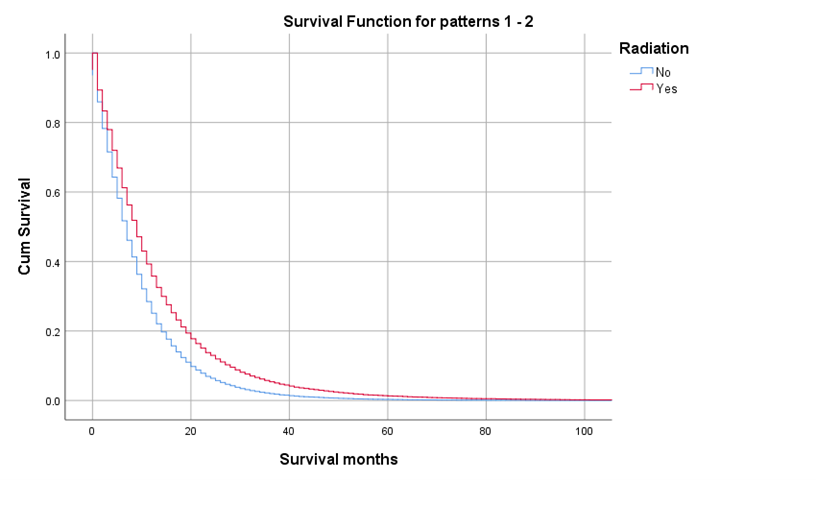


Fig.S10 Survival Curve for Cancer-Specific Mortality with Radiation.


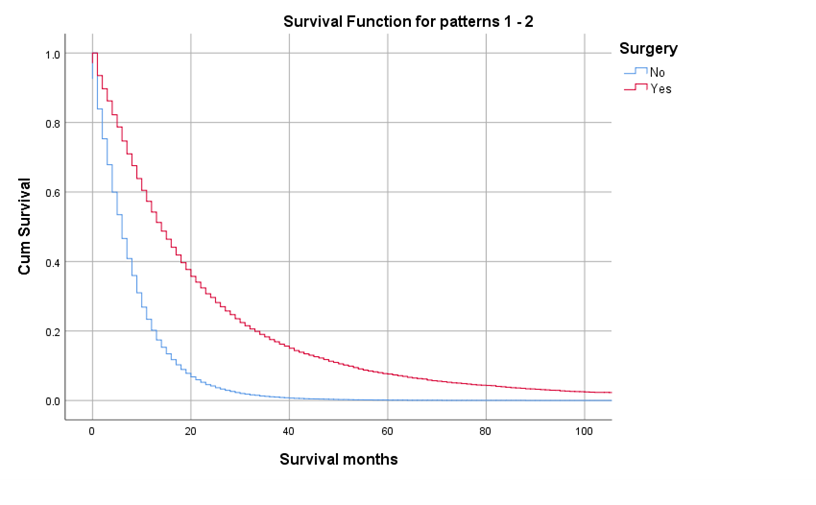


Fig.S11 Survival Curve for Cancer-Specific Mortality with Surgery.


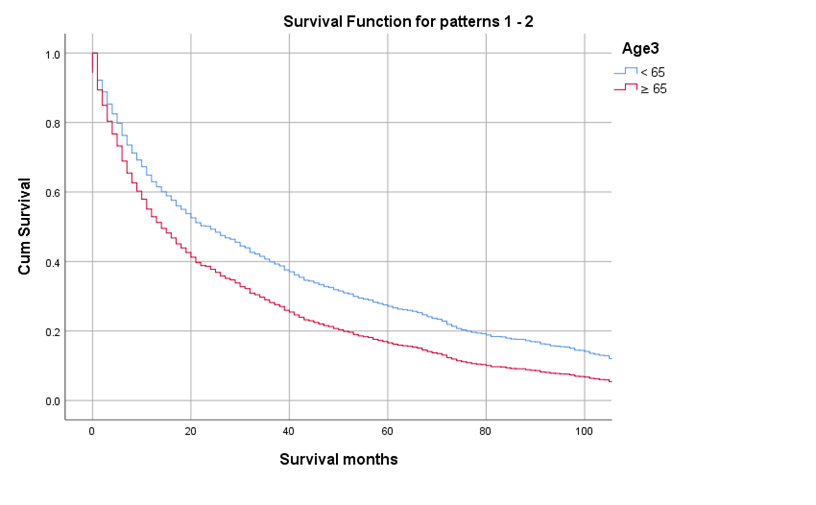


Fig.S12 Survival Curve for Cardiovascular Mortality with Age.


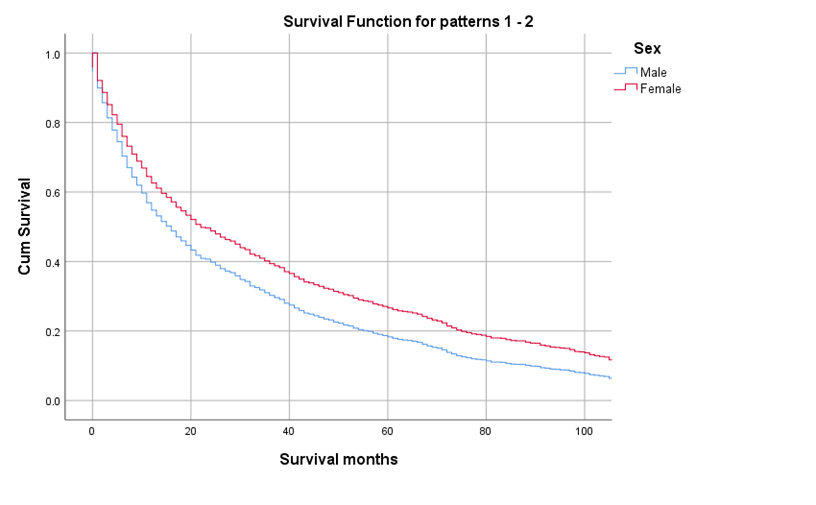


Fig.S13 Survival Curve for Cardiovascular Mortality with Sex.


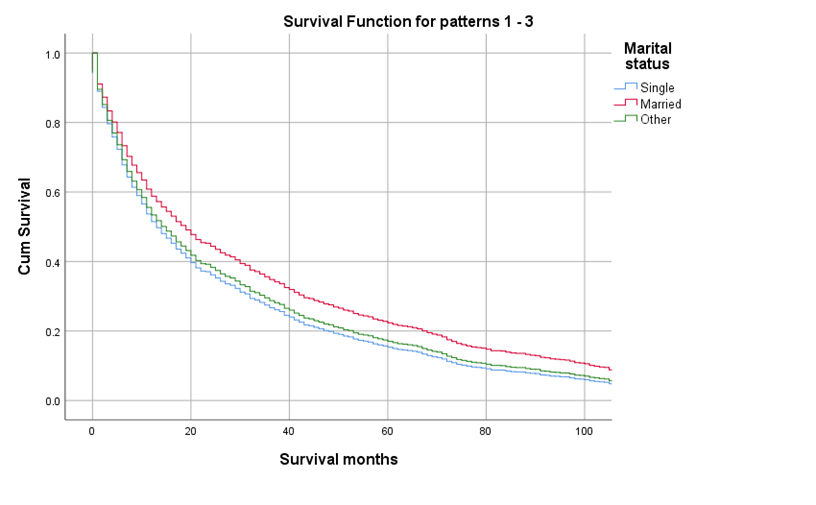


Fig.S14 Survival Curve for Cardiovascular Mortality with Marital status.


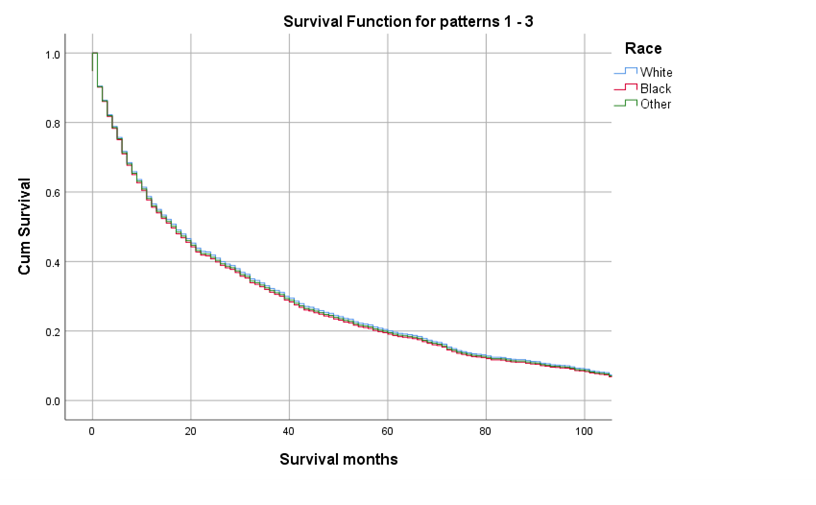


Fig.S15 Survival Curve for Cardiovascular Mortality with Race.


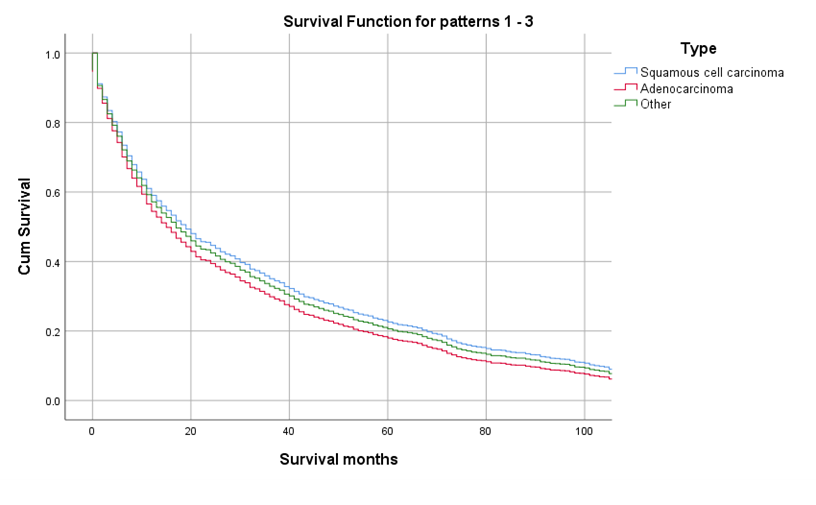


Fig.S16 Survival Curve for Cardiovascular Mortality with Type.


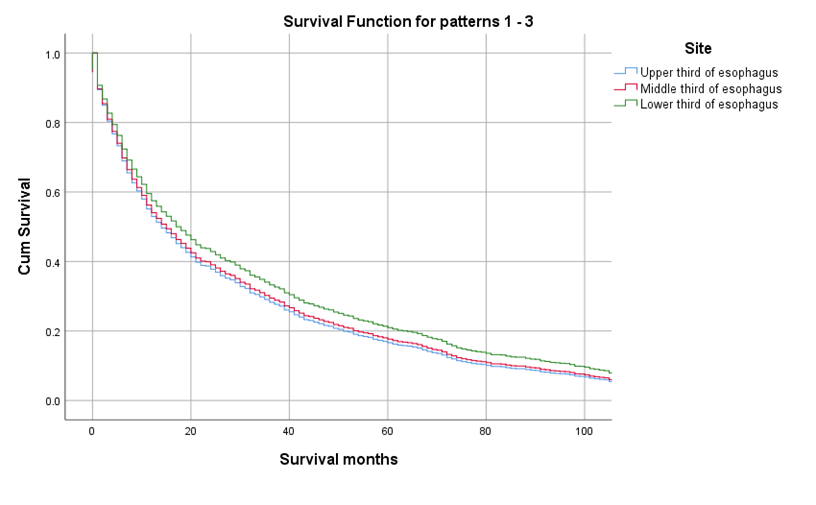


Fig.S17 Survival Curve for Cardiovascular Mortality with Site.


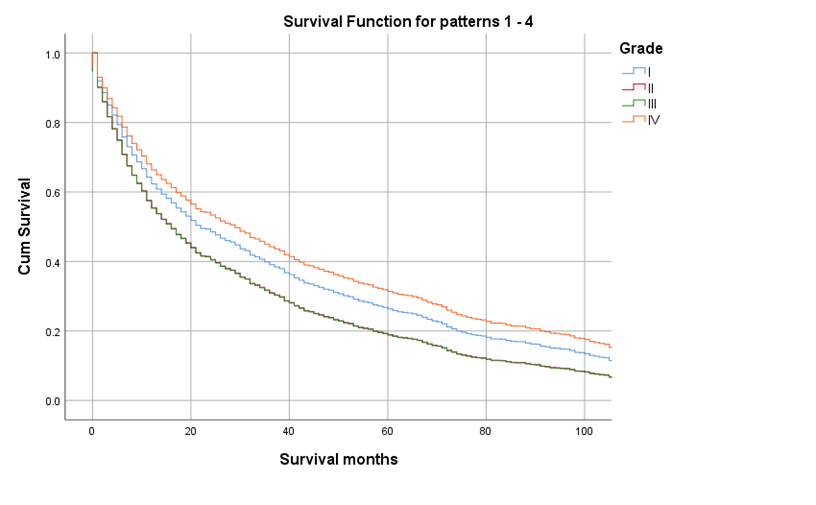


Fig.S18 Survival Curve for Cardiovascular Mortality with Grade.


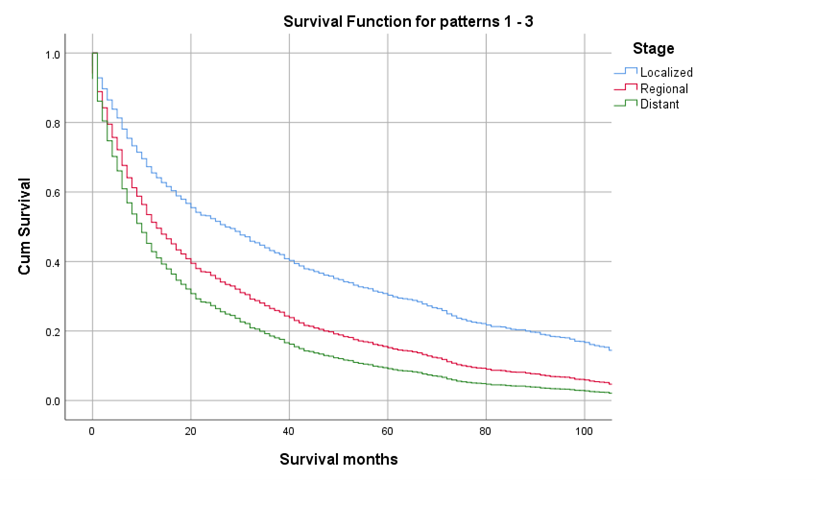


Fig.S19 Survival Curve for Cardiovascular Mortality with Stage.


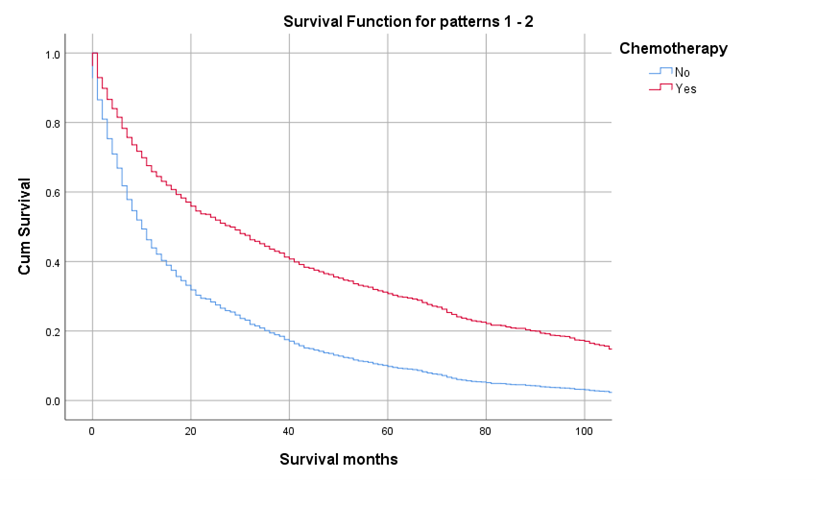


Fig.S20 Survival Curve for Cardiovascular Mortality with Chemotherapy.


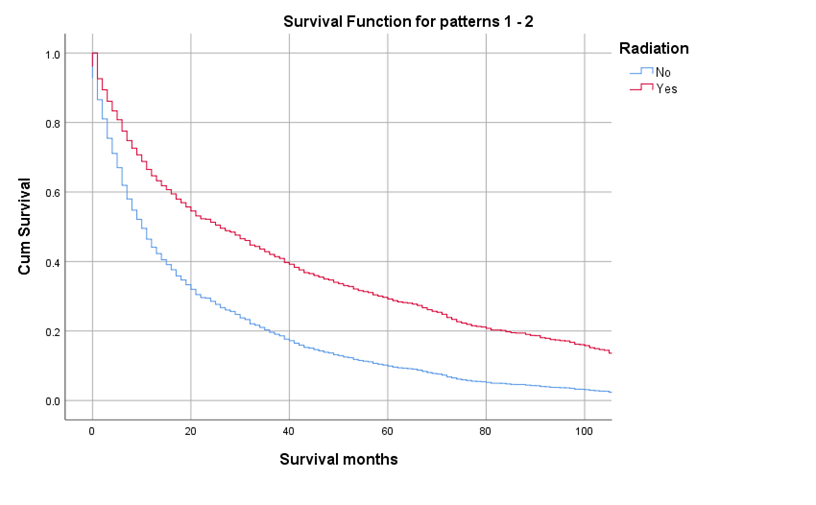


Fig.S21 Survival Curve for Cardiovascular Mortality with Radiation.


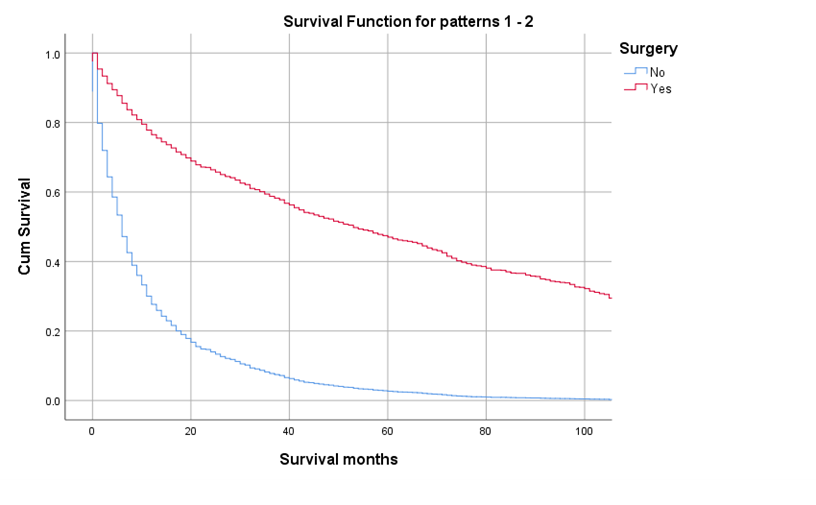


Fig.S22 Survival Curve for Cardiovascular Mortality with Surgery.
